# Lipidomics and Comparative Metabolite Excretion Analysis of Methanogenic Archaea Reveal Organism-Specific Adaptations to Varying Temperatures and Substrate Concentrations

**DOI:** 10.1128/msystems.01159-22

**Published:** 2023-03-07

**Authors:** Ruth-Sophie Taubner, Lydia M. F. Baumann, Michael Steiner, Kevin Pfeifer, Barbara Reischl, Kordian Korynt, Thorsten Bauersachs, Barbara Mähnert, Elisabeth L. Clifford, Jörn Peckmann, Bernhard Schuster, Daniel Birgel, Simon K.-M. R. Rittmann

**Affiliations:** a Archaea Physiology & Biotechnology Group, Department of Functional and Evolutionary Ecology, Universität Wien, Vienna, Austria; b Institute for Synthetic Bioarchitectures, Department of Bionanosciences, University of Natural Resources and Life Sciences, Vienna, Austria; c Institute for Chemical Technology of Organic Materials, Johannes Kepler Universität Linz, Linz, Upper Austria, Austria; d Space Research Institute, Austrian Academy of Sciences, Graz, Styria, Austria; e Institute for Geology, Center for Earth System Research and Sustainability, Universität Hamburg, Hamburg, Germany; f Arkeon GmbH, Tulln an der Donau, Austria; g Institute of Geosciences, Department of Organic Geochemistry, Christian Albrechts Universität, Kiel, Schleswig-Holstein, Germany; h Marine Biology/Microbial Oceanography, Department of Functional and Evolutionary Ecology, Universität Wien, Vienna, Austria; Woods Hole Oceanographic Institution

**Keywords:** anaerobes, microbial physiology, amino acids, isoprenoids, lipids, methane, *Archaea*, biotechnology, ecophysiology, methanogenesis

## Abstract

Methanogenic archaea possess diverse metabolic characteristics and are an ecologically and biotechnologically important group of anaerobic microorganisms. Although the scientific and biotechnological value of methanogens is evident with regard to their methane-producing physiology, little is known about their amino acid excretion, and virtually nothing is known about the lipidome at different substrate concentrations and temperatures on a quantitative comparative basis. Here, we present the lipidome and a comprehensive quantitative analysis of proteinogenic amino acid excretion as well as methane, water, and biomass production of the three autotrophic, hydrogenotrophic methanogens Methanothermobacter marburgensis, Methanothermococcus okinawensis, and Methanocaldococcus villosus under varying temperatures and nutrient supplies. The patterns and rates of production of excreted amino acids and the lipidome are unique for each tested methanogen and can be modulated by varying the incubation temperature and substrate concentration, respectively. Furthermore, the temperature had a significant influence on the lipidomes of the different archaea. The water production rate was much higher, as anticipated from the rate of methane production for all studied methanogens. Our results demonstrate the need for quantitative comparative physiological studies connecting intracellular and extracellular constraints of organisms to holistically investigate microbial responses to environmental conditions.

**IMPORTANCE** Biological methane production by methanogenic archaea has been well studied for biotechnological purposes. This study reveals that methanogenic archaea actively modulate their lipid inventory and proteinogenic amino acid excretion pattern in response to environmental changes and the possible utilization of methanogenic archaea as microbial cell factories for the targeted production of lipids and amino acids.

## INTRODUCTION

Methanogenic archaea (methanogens) are a phylogenetically and physiologically diverse group of anaerobic microorganisms (1–3). The main metabolic end product of their carbon and energy metabolism is methane (CH_4_), a major greenhouse gas and an important energy carrier. Methanogens are ecologically versatile in anoxic environments, consuming gaseous compounds and small organic molecules, thereby accelerating the breakdown of biomass. Due to their phylogenetic, evolutionary, ecological, and environmental importance, methanogens are model organisms for ecophysiological (2, 3), geobiological (4), astrobiological (3, 5, 6), and biotechnological (7–9) studies.

Although the scientific and biotechnological value of methanogens is evident with regard to their CH_4_-producing physiology (9–11), little is known about their amino acid excretion pattern, and virtually nothing is known about the lipidome at varying substrate concentrations and temperatures on a quantitative comparative basis. Both amino acids and isoprenoid-containing membrane core lipids are biotechnological products with increasing global demand (12, 13). In pure culture, methanogens are currently employed as CH_4_ cell factories (9, 11, 14–16). With regard to isoprenoids and amino acids, however, methanogens could become cell factories for the production of additional valuable bioproducts in combined gas, liquid, and solid production bioprocesses.

Proteinogenic amino acids are widely used in food and nutritional sciences, and especially, the fermentative production of amino acids is a well-established branch of biotechnology. To date, only specifically developed mutants or genetically engineered bacteria such as Corynebacterium glutamicum and Escherichia coli are used for that purpose (12). Although the uptake of amino acids in methanogens and their pathways for amino acid synthesis were investigated previously (17, 18), the ecophysiology of proteinogenic amino acid excretion has not yet been examined. Moreover, amino acid excretion by methanogens is still new ground in microbial ecophysiology. Amino acid excretion is relevant for the functioning of microbial ecosystems as, e.g., methanogens were recently implicated in providing amino acids as the substrate for subsequent fermentation by a syntrophic archaeal partner (19).

Various microorganisms are currently utilized for the biotechnological production of lipids, but these comprise mainly representatives of *Bacteria* and *Eukarya* (20–22). Although the lipid inventory of *Archaea* (including methanogens) and its potential application for biotechnology have been studied over the past decades (23–25), only a few biotechnological inventions, like archaeosomes (26), liposomes made from isoprenoidal lipids, are established. These are utilized, e.g., as durable lipid coatings with excellent biocompatibility (27).

Little information about changes in the lipid inventory of thermophilic methanogens in various environments is available to date (28, 29). A few lipid culture experiments have addressed such issues, e.g., for Methanocaldococcus jannaschii, a hyperthermophilic methanogen with an optimal growth temperature of ca. 85°C (30). M. jannaschii was cultured in a temperature range from 47°C to 78°C and showed a decrease in archaeol and concomitant increases in macrocyclic archaeol and GDGT-0 (glycerol dialkyl glycerol tetraether) with increasing temperatures (28). *M. jannaschii* was also grown under changing pressure and revealed an increase in macrocyclic archaeol and decreases in archaeol and GDGT-0 with increasing growth pressures (29). Methanothermobacter thermautotrophicus was grown under varying nutrient supplies, namely, varying molecular hydrogen (H_2_) levels and micronutrient availabilities (limitation of potassium and phosphate) (31).

Our study represents a comprehensive physiological analysis of three isolates of methanogens: Methanothermobacter marburgensis (order *Methanobacteriales*), a methanogen utilized for renewable energy production (9, 11) that was isolated from sewage sludge (32); Methanothermococcus okinawensis (*Methanococcales*), an organism with importance to biotechnology (11) and astrobiology (6, 33); and Methanocaldococcus villosus (*Methanococcales*), a hyperthermophilic and methanogenic model organism (6, 10). The latter two organisms were isolated from hydrothermal systems (34, 35).

The aim of this study was to perform a quantitative analysis of the lipidome and metabolite excretion patterns of these three methanogens in response to changing environmental conditions. Specifically, our experimental approach enabled us to compare and quantitatively link archaeal amino acid excretion patterns and lipid inventories to CH_4_, water (H_2_O), and biomass production as well as substrate and ammonium (NH_4_^+^) uptake rates in relation to different temperatures and substrate concentrations.

## RESULTS

**FIG 1:**
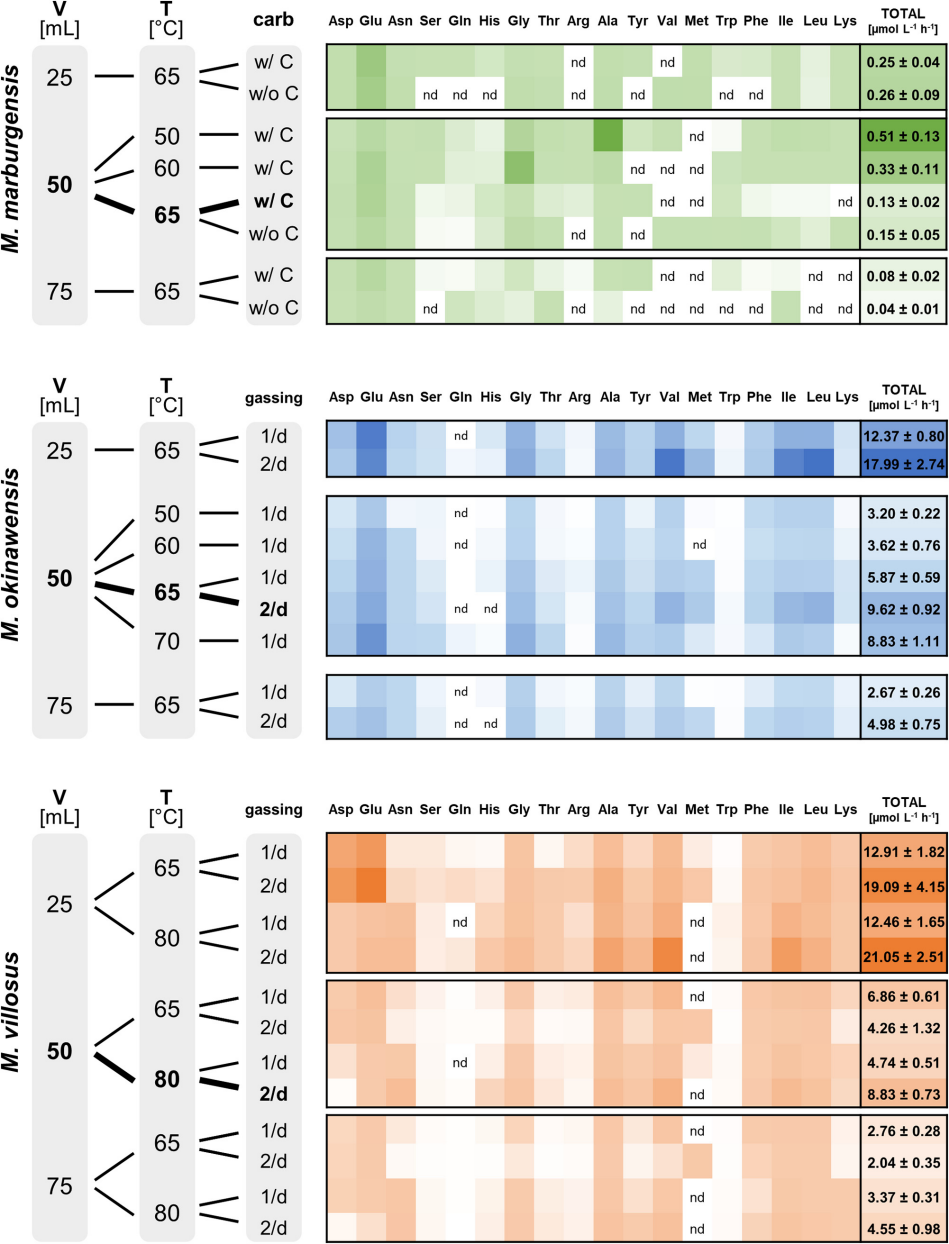
Mean amino acid excretion rates (AAERs) for *M. marburgensis* (green), *M. okinawensis* (blue), and *M. villosus* (orange). Shown are AAERs for different species in micromoles per liter per hour based on endpoint measurements. A darker color represents a higher number (total numbers added) (“nd” means no detection). The standard growth conditions are highlighted in boldface type. The medium for *M. marburgensis* (65°C) (with and without carbonates) and the gassing ratio for *M. okinawensis* (65°C) and *M. villosus* (65°C and 80°C) (once per day [1/d] and twice per day [2/d]) were varied. The liquid volume (V) varied between 25, 50, and 75 mL in a 120-mL serum bottle. *M. marburgensis* was always gassed once per day, and there were no changes in the compositions of the *M. okinawensis* and *M. villosus* media in this study. T, temperature.

**FIG 2:**
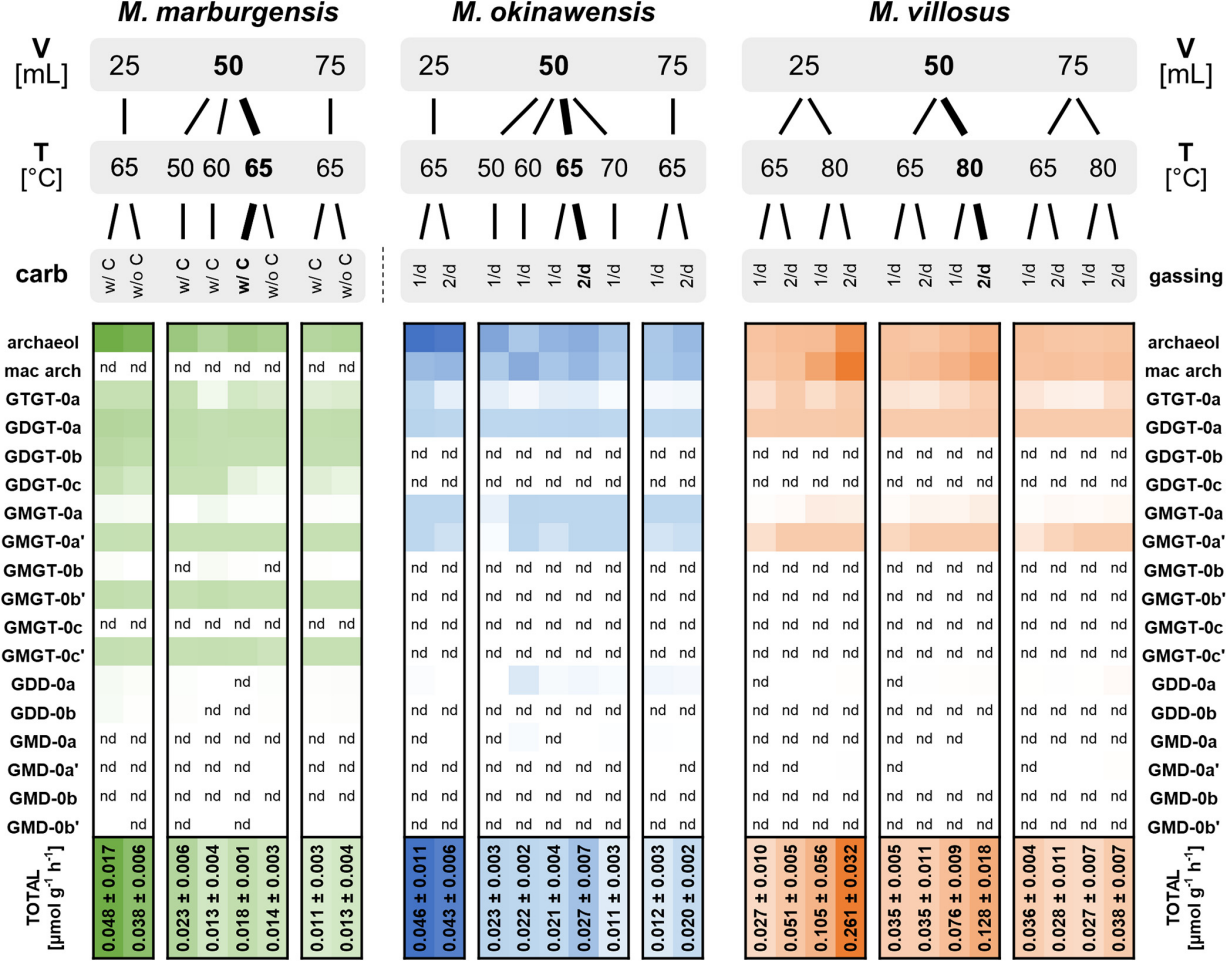
Mean lipid production rates for *M. marburgensis* (green), *M. okinawensis* (blue), and *M. villosus* (orange). Shown are lipid production rates for different species in micromoles per liter per hour based on endpoint measurements. A darker color represents a higher number (total numbers added) (“nd” means no detection). The standard growth conditions are highlighted in boldface type. The medium for *M. marburgensis* (65°C) (with and without carbonates) and the gassing ratio for *M. okinawensis* (65°C) and *M. villosus* (65°C and 80°C) (once per day [1/d] and twice per day [2/d]) were varied. The liquid volume (V) varied between 25, 50, and 75 mL in a 120-mL serum bottle. *M. marburgensis* was always gassed once per day, and there were no changes in the compositions of the *M. okinawensis* and *M. villosus* media in this study. T, temperature.

**FIG 3:**
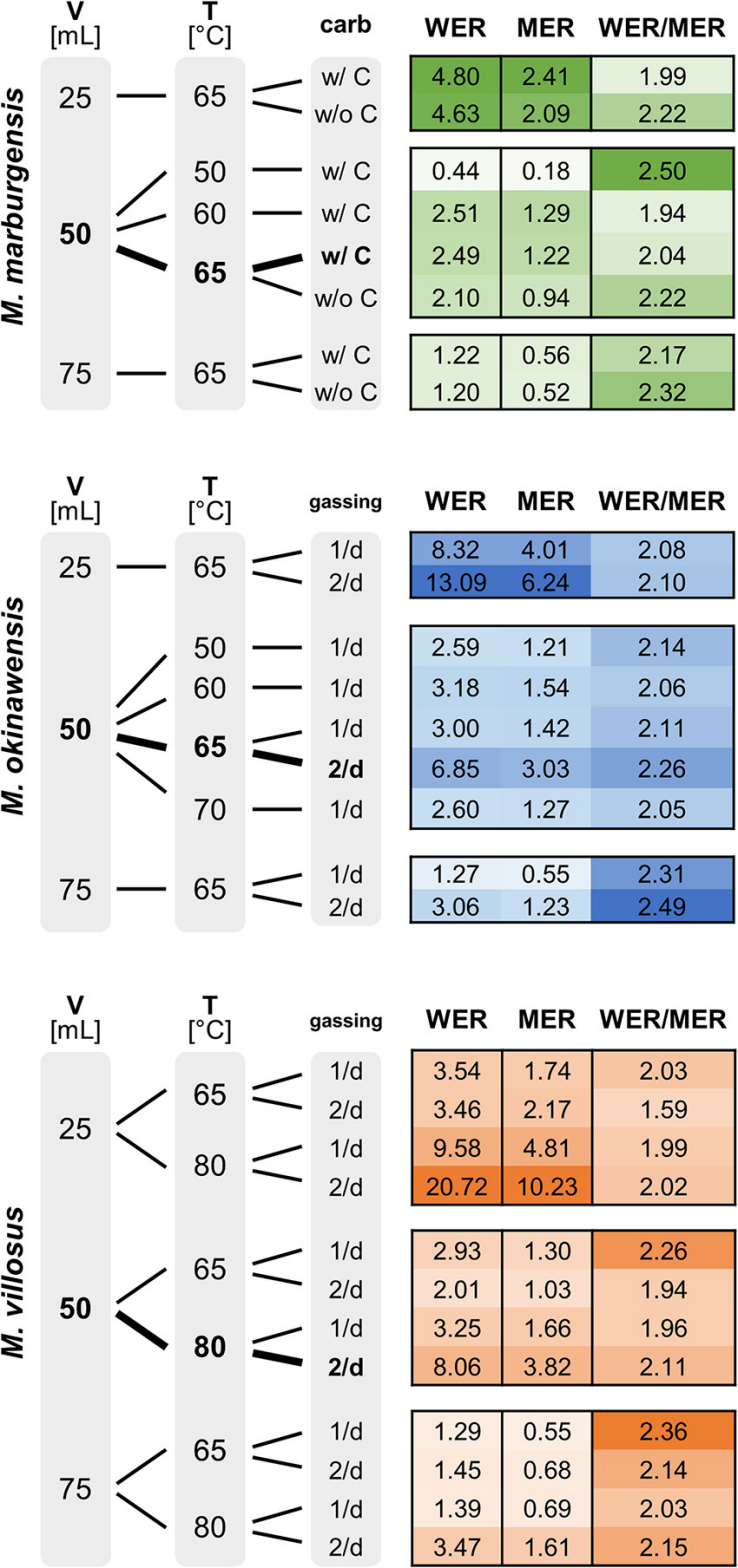
Mean CH_4_ evolution rate (MER) (millimoles per liter per hour), mean H_2_O evolution rate (WER) (millimoles per liter per hour), and WER/MER ratio for *M. marburgensis* (green), *M. okinawensis* (blue), and *M. villosus* (orange). The standard growth conditions are highlighted in boldface type. A darker color represents a higher number. The medium for *M. marburgensis* (65°C) (with and without carbonates) and the gassing ratio for *M. okinawensis* (65°C) and *M. villosus* (65°C and 80°C) (once per day [1/d] and twice per day [2/d]) were varied. The liquid volume varied between 25, 50, and 75 mL in a 120-mL serum bottle. *M. marburgensis* was always gassed once per day, and there were no changes in the compositions of the *M. okinawensis* and *M. villosus* media in this study.

The lipidome; amino acid excretion patterns; as well as CH_4_, H_2_O, and biomass production characteristics were investigated under varying environmental conditions, i.e., varying temperatures, gas-to-liquid ratios, gassing periods, and medium compositions, respectively. An overview of the different tested conditions is given in Fig. 1 to 3.

### Observed patterns under standard growth conditions.

For comparing the various strains with each other, the above-mentioned characteristics were first analyzed under standard growth conditions in a liquid volume of 50 mL (Fig. 1 to 3, left, marked in boldface type). Standard growth conditions are defined as growth at 65°C and a medium with carbonates for M. marburgensis, growth at 65°C and gassing two times per day for M. okinawensis, and growth at 80°C and gassing two times per day for M. villosus, all in a 50-mL volume (Fig. 1 to 3, marked boldface type, and Table 1).

**TABLE 1:** Growth, production, and excretion parameters for *M. marburgensis*, *M. okinawensis*, and *M. villosus* under standard growth conditions^*a*^

Parameter	Value for organism		
	*M. marburgensis*	*M. okinawensis*	*M. villosus*
*t*_inc_ (h)	229.3	78.6	36.9
Mean OD_end_ (at 578 nm) ± SD	1.041 ± 0.057	0.660 ± 0.040	0.700 ± 0.012
MER_mean_ (mmol L^−1^ h^−1^) ± SD	1.219 ± 0.076	3.030 ± 0.113	3.875 ± 0.237
WER_mean_ (mmol L^−1^ h^−1^) ± SD	2.485 ± 0.142	6.852 ± 0.180	8.056 ± 0.419
WER/MER ratio	2.038	2.262	2.115
Mean total AAER (μmol L^−1^ h^−1^) ± SD	0.125 ± 0.019	9.617 ± 0.916	8.831 ± 0.734
Mean total lipid production rate (μmol g^−1^ h^−1^) ± SD	0.018 ± 0.001	0.027 ± 0.007	0.128 ± 0.018

a *t*_inc_, total incubation time; OD_end_, maximal optical density (at 578 nm) measured over the total incubation time (available for only half of the samples); MER_mean_, mean methane evolution rate; WER_mean_, mean water evolution rate; AAER, amino acid excretion rate. Except for *t*_inc_ and the WER/MER ratio, the data presented are mean values and standard deviations (*n* = 4).

Even though *M. marburgensis* showed the highest optical density measured over the total incubation time (OD_end_), the mean methane evolution rate (MER_mean_), the mean water evolution rate (WER_mean_), the total amino acid excretion rate (AAER) (given in micromoles per liter per hour), and the total lipid production rate (given in nanomoles per gram per hour) of this strain were lower than those of the other two strains (Table 1 and Fig. 1 to 3). While Glu (0.086 ± 0.014 μmol L^−1^ h^−1^) is the dominating amino acid in *M. marburgensis* (almost 70% of the total amino acids), *M. okinawensis* shows the highest rates of production of Glu (1.567 ± 0.222 μmol L^−1^ h^−1^), Val (1.265 ± 0.202 μmol L^−1^ h^−1^), Ile (1.152 ± 0.139 μmol L^−1^ h^−1^), and Leu (1.337 ± 0.216 μmol L^−1^ h^−1^), and *M. villosus* shows the highest rates of production of Asn (1.211 ± 0.179 μmol L^−1^ h^−1^), Ala (1.203 ± 0.141 μmol L^−1^ h^−1^), Val (1.945 ± 0.157 μmol L^−1^ h^−1^), and Ile (1.382 ± 0.123 μmol L^−1^ h^−1^) (Fig. 1). The low lipid production rate is caused mainly by the longer total incubation time (division by a higher value). The archaeol production rates are high in all three strains (0.014 ± 0.001 μmol g^−1^ h^−1^ in *M. marburgensis*, i.e., 7% of the total lipids; 0.015 ± 0.003 μmol g^−1^ h^−1^ in *M. okinawensis*, i.e., 54% of the total lipids; and 0.049 ± 0.014 μmol g^−1^ h^−1^ in *M. villosus*, i.e., 39% of the total lipids), and in *M. okinawensis* and *M. villosus*, the rates of macrocyclic archaeol production are equal or even higher (0.014 ± 0.005 μmol g^−1^ h^−1^ in *M. okinawensis*, i.e., 41% of the total lipids, and 0.072 ± 0.004 μmol g^−1^ h^−1^ in *M. villosus*, i.e., 58% of the total lipids) (Fig. 2).

### Methanogens vary in their CH_4_/H_2_O product ratios.

H_2_O, as a basic requirement for life, is produced during hydrogenotrophic methanogenesis from H_2_ and CO_2_ according to the stoichiometry 4H_2_ + CO_2_ → CH_4_ + 2H_2_O (2). However, there is an enigma with regard to H_2_O production in methanogens, where a shift to higher rates of H_2_O than of CH_4_ production was detected in chemostat cultures of *M. marburgensis* (36). This prompted us to quantitatively investigate the H_2_O/CH_4_ product ratio, i.e., the ratio of WER/MER, of the three strains with respect to varying substrate concentrations and temperatures.

The MERs for each experimental setup are shown in Fig. 3. The MER was higher for those experiments with a larger headspace volume, i.e., a smaller liquid volume (“25 mL”). In numbers, the ratios of 25/50/75 mL (without optical density [OD] measurements) under standard growth conditions were approximately 4.3:2.2:1 for *M. marburgensis*, approximately 5.1:2.5:1 for *M. okinawensis*, and 6.3:2.4:1 for *M. villosus*. Minor MER variations were observed for *M. marburgensis* for experiments performed with or without carbonate in the medium. The highest MERs for *M. okinawensis* and *M. villosus* were observed at their optimal growth temperatures (65°C and 80°C, respectively) at 25 mL with twice-per-day gassing (6.24 ± 0.55 mmol L^−1^ h^−1^ for *M. okinawensis* and 10.23 ± 0.79 mmol L^−1^ h^−1^ for *M. villosus*). The same pattern was observed for the WER determined via mass increases (see reference 10 for detailed information) in an isobaric setting (Fig. 3).

Theoretically, H_2_O-CH_4_ (and, consequently, the WER and MER) should correspond to a 2:1 ratio. However, a shift toward a WER/MER ratio of >2 was observed for the majority of cultures in a closed batch mass balance setting (Fig. 3). A trend toward a ratio of <2 was observed for *M. villosus* grown in a 25-mL liquid volume (65°C) with twice-per-day gassing. Moreover, a change in the WER for *M. okinawensis* at various incubation temperatures and gassings per day occurred, whereby twice-per-day gassing led to a higher WER. Gassing twice per day at 80°C resulted in an increase in the WER for *M. villosus*. In total volumes, from one gassing event to the other, *M. marburgensis* produced on average 46.5 ± 2.0 μL H_2_O, *M. okinawensis* produced 51.9 ± 1.9 μL H_2_O, and *M. villosus* produced 50.5 ± 2.2 μL H_2_O (under their respective standard growth conditions).

### Methanogens modulate amino acid excretion patterns and rates.

Besides the investigation of the CH_4_/H_2_O product ratio, this study also presents a quantitative determination of the proteinogenic amino acid excretion patterns of the three methanogenic strains. To demonstrate that amino acids were actively excreted and not associated with cell lysis, an experiment was designed where once-per-day OD measurements were combined with daily amino acid and fluorescence-activated cell sorting (FACS) measurements (see Fig. S1 and S2 in the supplemental material). These experiments were performed by using an initial liquid volume of 50 mL under the respective standard growth conditions of the three strains (once-per-day gassing for *M. okinawensis*). As *M. villosus* showed the highest rate of cell lysis of the three methanogens, we additionally determined the level of amino acids released due to cell lysis by continuing the incubation of the culture in stationary phase without the addition of gaseous substrates. After approximately 113 h of incubation without additional gassing, the total biomass had decreased from 0.30 g L^−1^ to 0.06 g L^−1^ (i.e., a decrease of 82%; viable biomass, 0.29 g L^−1^ to 0.01 g L^−1^) (Fig. S2c), an indication of cell death and lysis. While the total amino acid content increased by only 25% in that period, an increase of 440% during exponential cell growth within the first 64 h of the experiment was observed (Fig. S2). These findings confirm our hypothesis that amino acids are actively excreted at a higher rate during exponential growth than during cell lysis.

10.1128/msystems.01159-22.1 FIG S1 Examples of gating for the analysis of flow cytometry data for Syto9- and propidium iodide-stained samples of Methanothermobacter marburgensis, Methanothermococcus okinawensis, and Methanocaldococcus villosus cultures. One milliliter of the growing culture was sampled once a day. Twenty microliters of the sample was diluted in 400 μL of minimal medium of the respective organism, stained with Syto9 and propidium iodide (PI) (Live/Dead BacLight bacterial viability kit; Invitrogen), and analyzed (FACSCanto II; BD). A total of 10,000 events were gated according to forward scattering (FSC) and side scattering (SSC) and fluorescence (Syto9, FITC; PI, PerCP-Cy5-5H) after fluorescence compensation was performed on the FITC and PerCP-Cy5-5H channels. The zero controls were gated in the same manner, and the recorded events were subtracted from each sample. Download FIG S1, PDF file, 0.1 MB. Copyright © 2023 Taubner et al. 2023 Taubner et al. https://creativecommons.org/licenses/by/4.0/ This content is distributed under the terms of the Creative Commons Attribution 4.0 International license.

10.1128/msystems.01159-22.2 FIG S2 Total amino acid content, dead/live biomass content, and NH_4_^+^ uptake of Methanothermobacter marburgensis, Methanothermococcus okinawensis, and Methanocaldococcus villosus. Graphs show the concentrations of viable biomass (green line) and unviable biomass (red line) in grams per liter as determined by flow cytometry, the concentration of amino acids in the supernatant in micromoles per liter (yellow line) as determined by HPLC analysis, and the NH_4_^+^ uptake rate (blue line) in micromoles per liter measured with a plate photometer. The standard deviation of each measurement is based on the results of 4 biological replicates (50 mL at the respective optimal growth temperatures) with 3 technical replicates each time (*n* = 12). The cultures of *M. villosus* were gassed the last time at the 47-h time point (marked by the red dashed line) to determine amino acid excretion during extreme lysis (visible by the decrease in the viable biomass after 64 h). Download FIG S2, PDF file, 0.1 MB. Copyright © 2023 Taubner et al. 2023 Taubner et al. https://creativecommons.org/licenses/by/4.0/ This content is distributed under the terms of the Creative Commons Attribution 4.0 International license.

Interestingly, the amino acid excretion patterns varied over time. While the total number of amino acids steadily increased over time for the other two strains, *M. marburgensis* showed a peak at the 48.1-h time point (Fig. S2a). After a decrease for the next ca. 40 h, the total number of amino acids increased again until the end of the experiment. Within the first 48 h, Ser, Gly, Arg, Ala, Val, Met, Leu, and Ile reached their highest values. While Val, Met, and Ala dominated the amino acid pool until the 48.1-h time point, Glu was predominant thereafter. Changes in production were also observed for Glu in *M. okinawensis* (highest values at 90.3 h) and for Glu, Tyr, Met, and Trp in *M. villosus*.

**FIG 4:**
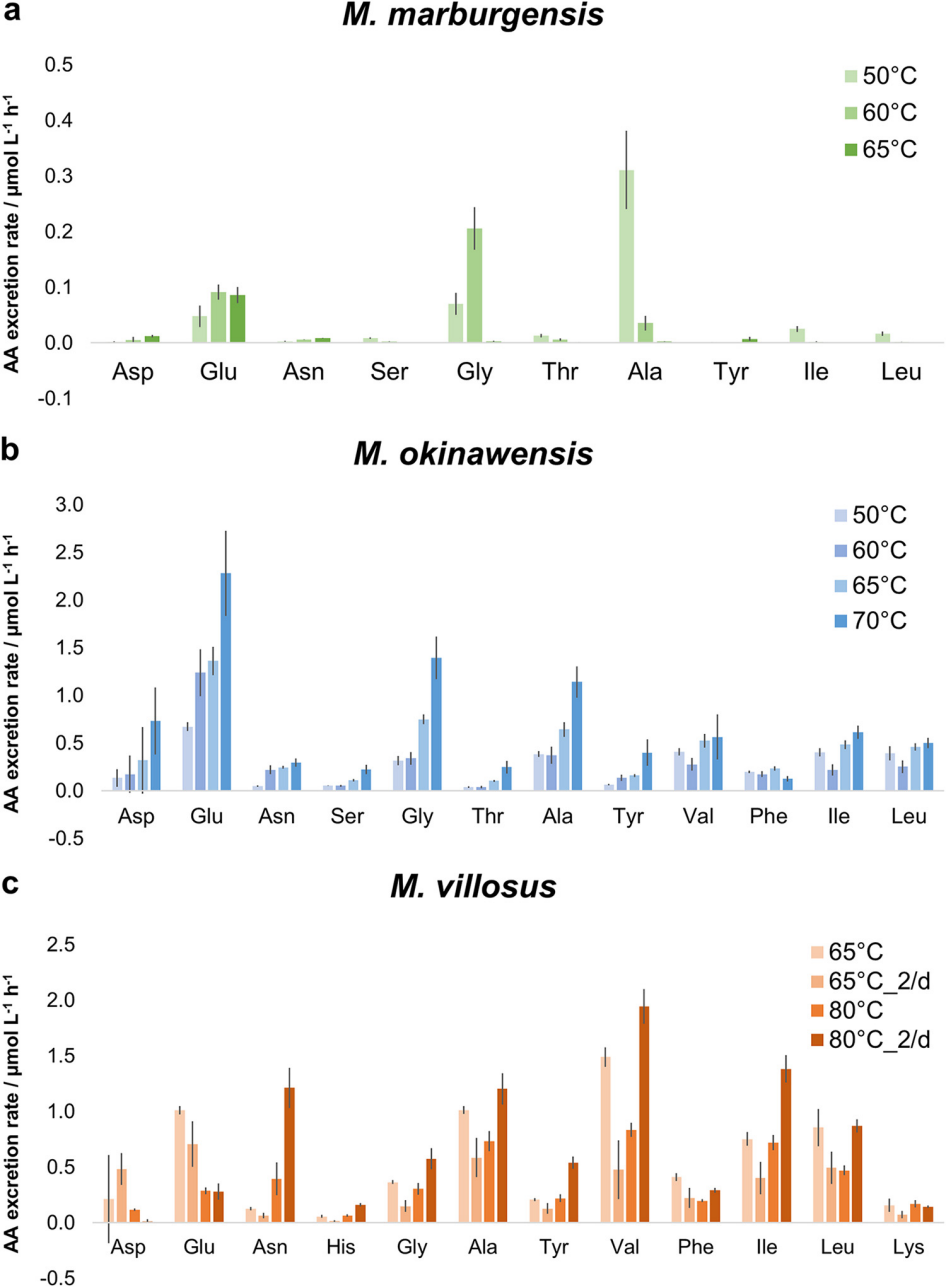
Amino acid (AA) excretion rates (AAERs) for *M. marburgensis*, *M. okinawensis*, and *M. villosus* at different temperatures (in a 50-mL initial liquid volume). Shown are AAERs for selected species in micromoles per liter per hour based on endpoint measurements for *M. marburgensis* (50°C, 60°C, and 65°C, with carbonates) (a), *M. okinawensis* (50°C, 60°C, 65°C, and 70°C, with once-per-day gassing) (b), and *M. villosus* (65°C and 80°C) (c). Each bar represents a set of octuplicates. The error bars indicate standard deviations. Experiments were performed at different incubation temperatures. “2/d” refers to two gassing procedures per day; otherwise, gassing was performed once per day.

At least traces of all of the 18 tested amino acids were found in most cultures at the end of the experiments. Figure 1 demonstrates that increased gaseous substrate availability was accompanied by increased amino acid excretion at the optimal growth temperature for each organism. In numbers, the ratios of 25/50/75 mL (without OD measurements) under standard growth conditions were approximately 3.2:1.6:1 for *M. marburgensis*, approximately 3.6:1.9:1 for *M. okinawensis*, and 4.6:1.9:1 for *M. villosus*. However, *M. marburgensis* excreted far fewer amino acids than the other two strains at the same substrate concentration and gassing interval (0.13 ± 0.02 μmol L^−1^ h^−1^ compared to 5.87 ± 0.59 μmol L^−1^ h^−1^ for *M. okinawensis* and 4.74 ± 0.51 μmol L^−1^ h^−1^ for *M. villosus* at the optimal growth temperature, with once-per-day gassing) (Fig. 1). The most prominent excreted amino acid in most *M. marburgensis* cultures at the optimal growth temperature (65°C) was Glu (up to 84% of the total measured amino acids in some cultures). Therefore, the observed decrease in the AAER in *M. marburgensis* from 25 mL to higher liquid volumes is caused mainly by the reduced production of Glu. Interestingly, the total AAER of *M. marburgensis* was higher at lower temperatures (50°C and 60°C) in a liquid volume of 50 mL (50°C, 0.51 ± 0.13 μmol L^−1^ h^−1^; 60°C, 0.33 ± 0.11 μmol L^−1^ h^−1^; 65°C, 0.13 ± 0.02 μmol L^−1^ h^−1^), which was also observed for *M. villosus* for the once-per-day gassing experiments in 25 and 50 mL and for *M. okinawensis* for the experiments performed at 70°C (Fig. 1 and 4). This characteristic of *M. marburgensis* at 50°C is due to an extreme increase in the rates of excretion of Ala (from 2.7 ± 0.5 nmol^−1^ L^−1^ h^−1^ at 65°C to 311 ± 70 nmol^−1^ L^−1^ h^−1^ at 50°C) and Gly (from 2.9 ± 0.9 nmol^−1^ L^−1^ h^−1^ at 65°C to 206 ± 38 nmol^−1^ L^−1^ h^−1^ at 60°C) (Fig. 4). The high AAER of *M. okinawensis* at 70°C is caused mainly by increases in Glu, Gly, and Ala (Fig. 4).

*M. okinawensis* showed the highest total AAER under its standard growth conditions (9.62 ± 0.92 μmol L^−1^ h^−1^), which agrees with the kinetics of substrate uptake (10). However, the total AAER at 70°C was almost as high as that under the standard growth conditions, caused by extreme increases in the rates of production of Glu, Gly, and Ala (Fig. 4). In general, amino acid patterns varied at different growth temperatures (Fig. 4). No interpretation of the Asp pattern is possible as it was detected in only approximately one-half of the replicates (resulting in high error bars) (Fig. 4).

The total AAER of *M. villosus* was in the same range as that of *M. okinawensis* (Fig. 1). However, the patterns of the individual amino acids were different between the two strains (Fig. 1 and 4). For *M. villosus*, Val, Ile, Ala, Asn, and Leu were the most prominent amino acids under standard growth conditions, while for *M. okinawensis*, Glu and Gly replaced Asn as the dominant amino acids. A different pattern was found for *M. villosus* at a lower temperature (65°C) for the 25-mL samples, representing the only setting where Asp (3.82 ± 1.32 μmol L^−1^ h^−1^, with twice-per-day gassing) and Glu (5.66 ± 1.92 μmol L^−1^ h^−1^, with twice-per-day gassing) not only dominated but also showed the highest rates in all experiments performed in this study (Fig. 1). At 65°C, gassing once per day (50 mL) (Fig. 1 and 4) promoted higher AAERs of *M. villosus* than the in twice-per-day gassing experiments, which was unexpected (and was not observed at 80°C) as it has been suggested that higher substrate (gas) availability leads to the higher-level production of metabolites (10). At 80°C with a 50-mL volume, twice-per-day gassing led to higher rates of excretion of mainly Val (1.94 ± 0.16 μmol L^−1^ h^−1^), Asn (1.21 ± 0.18 μmol L^−1^ h^−1^), Ile (1.38 ± 0.12 μmol L^−1^ h^−1^), Ala (1.20 ± 0.14 μmol L^−1^ h^−1^), and Leu (0.87 ± 0.06 μmol L^−1^ h^−1^) in *M. villosus* (Fig. 4). Noteworthy, Met excretion rates were observed only for cultures gassed twice per day at 65°C (for all volumes).

A comparison of the NH_4_^+^ uptake rate and the AAER (both in micromoles per liter per hour) revealed a ratio close to 1:1 in *M. okinawensis.* For *M. marburgensis*, substantially more NH_4_^+^ was taken up than amino acids were excreted (Fig. S3). In the course of the lysis experiments, it was shown that the extreme NH_4_^+^ uptake by *M. marburgensis* mainly took place immediately before the strain entered the stationary growth phase (Fig. S2a, blue line). On the contrary, the AAER in *M. villosus* was higher than the NH_4_^+^ uptake rate (Fig. S3).

10.1128/msystems.01159-22.3 FIG S3 Ammonium (NH_4_^+^) uptake rate versus amino acid excretion rate for *M. marburgensis*, *M. okinawensis*, and *M. villosus*. Amino acid excretion rates for different species and NH_4_^+^ uptake rates (both in micromoles per liter per hour) are based on endpoint measurements. Each data point represents a single replicate for the respective experiments (both with and without OD measurements). For *M. marburgensis*, the left and the middle panels represent experiments with varying liquid volumes (25, 50, and 75 mL) at the optimal growth temperature (65°C). The right panel represents experiments with a volume of 50 mL at different incubation temperatures (50°C, 60°C, and 65°C). The orange data points in the left and right panels are from the same experiment. For *M. okinawensis*, the left and the middle panels represent experiments with varying volumes (25, 50, and 75 mL) at the optimal growth temperature (65°C). The right panel represents experiments with a volume of 50 mL at different incubation temperatures (50°C, 60°C, 65°C, and 70°C). The orange data points in the left and right panels are from the same experiment. For *M. villosus*, the first two panels represent experiments with varying volumes (25, 50, and 75 mL) at 65°C, and the third and fourth panels represent experiments at the optimal growth temperature (80°C). For *M. marburgensis*, experiments were additionally performed without the use of carbonate (labeled “w/oC”). “2/d” refers to two gassing procedures per day; otherwise, gassing was performed once per day. Download FIG S3, PDF file, 0.2 MB. Copyright © 2023 Taubner et al. 2023 Taubner et al. https://creativecommons.org/licenses/by/4.0/ This content is distributed under the terms of the Creative Commons Attribution 4.0 International license.

### Lipidomes are affected by varying substrate concentrations and temperatures.

We compared the changes in the lipidomes of the three methanogens at different temperatures and substrate concentrations to reveal their lipid-specific adaptation patterns. The lipid inventory comprised the tetraether lipids GTGT-0 (glycerol trialkyl glycerol tetraether), GDGT-0 (glycerol dialkyl glycerol tetraether), and GMGT-0 (glycerol monoalkyl glycerol tetraether) as well as the diethers archaeol and macrocyclic archaeol (for chromatograms, see reference 37). For *M. marburgensis*, the degrees of methylation (0, 1, or 2) of the basic structures of GMGT-0 and GDGT-0 are displayed as the relative percentages of the different methylated species (a, b, or c) of the sum of all di- and tetraethers, respectively. The tetraether GMGT-0 comes as two isomers, one eluting earlier and the other eluting later. For the various strains, later-eluting isomers are indicated as GMGT-0a′, GMGT-0b′, and GMGT-0c′, respectively.

The most prominent lipids of *M. marburgensis* are archaeol, GDGT-0a, and GDGT-0b, whereas *M. okinawensis* and *M. villosus* predominantly synthesize archaeol, macrocyclic archaeol, and GDGT-0a (Fig. 2). A positive correlation between the level of gaseous substrates and the lipid production rate was observed (Fig. 2). Under standard growth conditions, the total lipid production rate for *M. marburgensis* added up to approximately 17.7 ± 1.2 nmol g^−1^ h^−1^, of which diether lipids (archaeol) made up more than 75%.

Under standard growth conditions, *M. villosus* revealed total lipid production rates up to seven times higher than the production rates of *M. marburgensis* and *M. okinawensis* (Fig. 2). While twice-per-day gassing resulted in higher rates of lipid production by *M. villosus* at the optimal growth temperature, no clear impact of the gassing rate on the lipid production rate in *M. okinawensis* was observed. Under standard growth conditions, *M. okinawensis* exhibited a mean total lipid production rate of 27.4 ± 7.0 nmol g^−1^ h^−1^, while that of *M. villosus* was 127.7 ± 18.5 nmol g^−1^ h^−1^. For both strains, mainly archaeol and macrocyclic archaeol were detected in these experimental settings (more than 95% of the total lipids).

With a volume of 25 mL, gassing twice per day resulted in a higher lipid production rate, while with a volume of 75 mL, the gassing rate showed no clear impact on the lipidome of *M. villosus* (Fig. 2).

**FIG 5:**
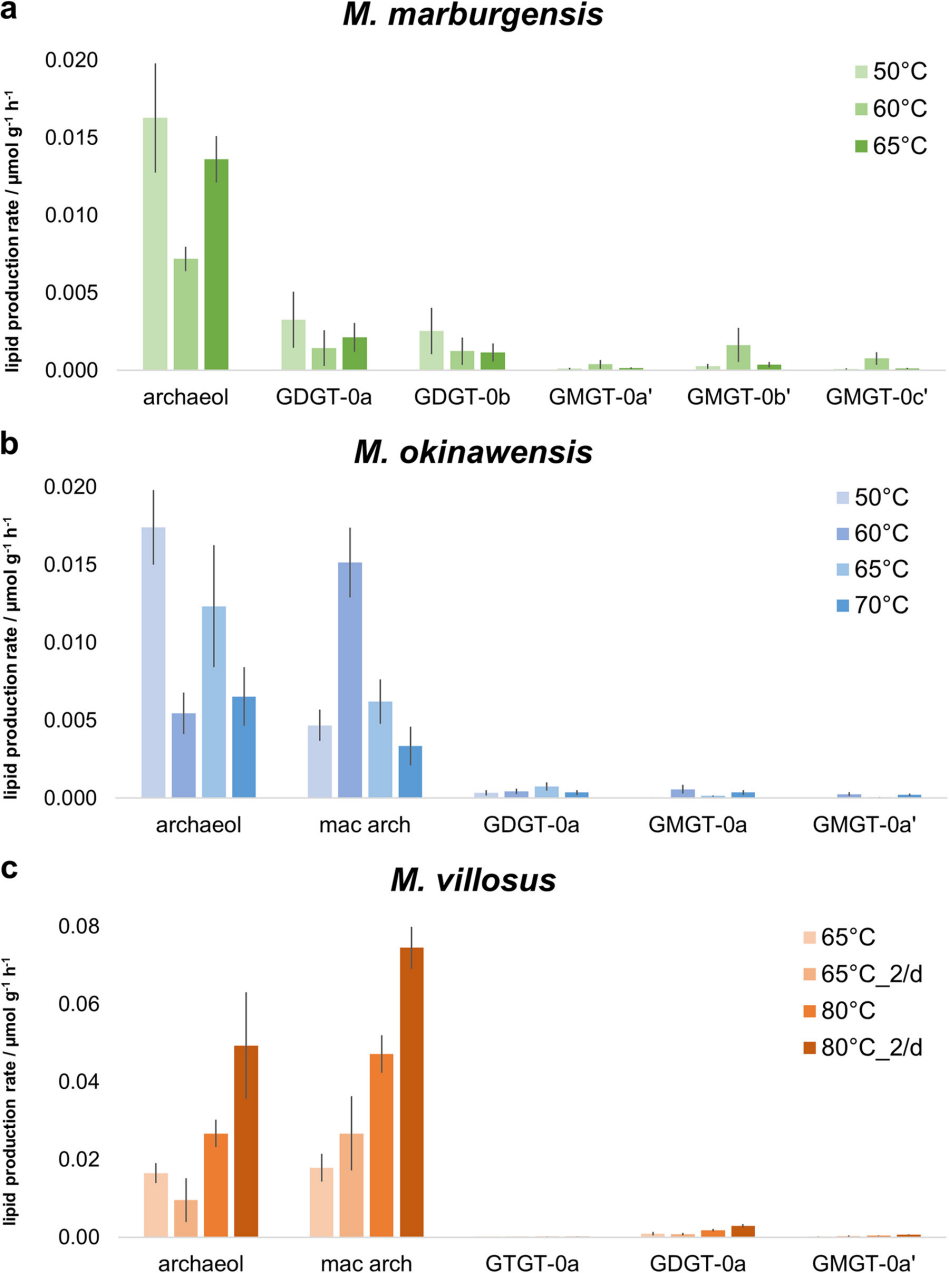
Lipid production rates for *M. marburgensis*, *M. okinawensis*, and *M. villosus* at different temperatures (in a 50-mL initial liquid volume). Shown are lipid production rates for selected species in micromoles per gram per hour based on endpoint measurements for *M. marburgensis* (50°C, 60°C, and 65°C, with carbonates) (a), *M. okinawensis* (50°C, 60°C, 65°C, and 70°C, with once-per-day gassing) (b), and *M. villosus* (65°C and 80°C) (c). Each bar represents a set of octuplicates. The error bars indicate standard deviations. Experiments were performed at different incubation temperatures. “2/d” refers to two gassing procedures per day; otherwise, gassing was performed once per day. mac arch, macrocyclic archaeol.

Variations in the temperature affected the lipid inventories of the three methanogens (Fig. 2 and 5). For *M. marburgensis*, the specific archaeol production rate in 50 mL liquid medium was high at 50°C (16.3 ± 3.5 nmol g^−1^ h^−1^), decreased at 60°C (7.2 ± 0.8 nmol g^−1^ h^−1^), and increased again at 65°C (13.6 ± 1.5 nmol g^−1^ h^−1^), while the total lipid production rate stayed almost constant at all temperatures (Fig. 2). A similar, but slightly weaker, trend was found for GDGT-0a and GDGT-0b compared to archaeol (Fig. 5). However, the specific GMGT-0b′ production rate increased only at 60°C (Fig. 5). When comparing the results of 50-mL experiments at 50°C, 60°C, and 65°C, the total specific lipid production rate stayed constant in *M. okinawensis* (Fig. 2), but the ratio between the specific archaeol and macrocyclic archaeol production rates changed (Fig. 5). *M. okinawensis* revealed a high specific archaeol production rate at 50°C and 65°C, while at 60°C, macrocyclic archaeol dominated. A decrease in the specific total lipid production rate was observed at 70°C for *M. okinawensis* at 50 mL (Fig. 2 and Fig. 5b). For *M. villosus*, the total specific lipid production rate was higher at 80°C than at 65°C (Fig. 2), and macrocyclic archaeol always dominated over archaeol in the 50-mL experiments (Fig. 5). At 25 and 75 mL, this trend was not observed.

## DISCUSSION

The aim of this study was to gain comprehensive insights into the lipidomes and metabolite excretion patterns of three methanogens and to report the environmental conditions under which these organisms actively modulate the CH_4_/H_2_O product ratio, amino acid excretion, and their lipidome as a function of substrate availability and temperature. Examining the changes in these interdependent variables presents an unprecedented attempt to holistically understand how the three strains physiologically respond to environmental alterations at the levels of gases and excreted liquid-phase compounds and by modulating their cytoplasmatic membrane components.

### Amino acid excretion by methanogens strongly depends on temperature and other variables.

To date, studies dealing with amino acids in methanogens are limited to the uptake of amino acids from the environment and the biochemistry of amino acid production pathways (38–40). For example, amino acid uptake, amino acid catabolism, and the pathway for Ile biosynthesis were studied in Methanococcus voltae (41). This methanogen incorporated different amino acids from the growth medium into its protein structures. In Methanothermobacter thermautotrophicus (42, 43) and *M. marburgensis* (44), amino acids are early fixation products of CO_2_ and NH_4_^+^ assimilation. Moreover, the effect of H_2_, Leu, or phosphate limitation on the free intracellular amino acid pools (and growth rates) of a Leu auxotroph mutant of Methanococcus maripaludis S2 was investigated. H_2_ limitation resulted in a decrease in the free intracellular amino acid levels, with Gly being an exception, increasing 2-fold during H_2_ limitation (45). We found that during H_2_ limitation (75 mL compared to 25 mL and 50 mL), the AAERs of Gly and all other amino acids are lower in all three tested strains (Fig. 1). To the best of our knowledge, the only study reporting the excretion of amino acids by methanogens used U-^14^C labeling to quantify the contribution of the endogenous synthesis of Leu and Ile compared to Leu and Ile uptake in M. voltae. Increases in the Leu and Ile concentrations in the supernatant were detected, which demonstrated that excess amino acids were excreted during growth (46).

By varying the growth temperature (at a constant volume), we were able to identify conclusive shifts in the amino acid patterns. These variations might be adaptations to environmental stressors or might reflect intercellular communication signals between different species. The latter was observed in a study where “*Candidatus* Prometheoarchaeum syntrophicum” strain MK-D1 was enriched together with a *Methanogenium* archaeon (19). “*Ca.* Prometheoarchaeum syntrophicum” degraded 10 different amino acids, providing H_2_ and/or formate to the *Methanogenium* archaeon in return, and received amino acids for its catabolism. That study and our study show that the ecophysiological interactions of microbes in enrichment cultures and natural environments, as well as their metabolic capabilities, are poorly understood on a qualitative and quantitative basis. To determine the syntrophic benefit of amino acid excretion for methanogens, further studies coupling *in vitro* and *in silico* physiologies will be of special interest to microbial ecophysiology, biotechnology, astrobiology, and beyond.

A key finding of our study is the higher AAER in *M. villosus* at 50 mL for cultures gassed once per day at 65°C than for those gassed twice per day. We hypothesize that under these specific growth conditions, *M. villosus* benefits from a longer, undisturbed incubation period. Furthermore, the AAERs of Asp and Glu in *M. villosus* at 25 mL at 65°C were higher than those in all other settings, and *M. okinawensis* showed extreme increases in the AAERs of Glu, Gly, and Ala for the experiments performed at 70°C, i.e., 5°C above the optimal growth temperature, in comparison to the experiments performed at lower temperatures (Fig. 4). These surprising findings should motivate future comprehensive studies to determine the optimal conditions for high AAERs for the different amino acids.

### The lipidome is less dependent on taxonomy than on environmental conditions.

Numerous studies have examined the lipid biomarker inventory of methanogens and other, nonmethanogenic organisms under changing temperatures, pressures, and pH (28, 47, 48). A general outcome of these studies was, among others, that diether lipid production seems to be a membrane lipid adaption to lower temperatures and higher pressures, with regard to the optimal growth conditions, and vice versa for the production of tetraether membrane lipids. It has to be noted that most experimental results are available for thermophiles, such as the archaea chosen for this study. A recent study on the piezo-hyperthermophilic archaeon Thermococcus barophilus already confirmed that tetraether lipids were increased with low pressure and high temperatures, whereas archaeol was produced at low temperatures and high pressure (49). In *M. villosus* and *M. okinawensis*, we additionally monitored the production of macrocyclic archaeol, which is a unique and only rarely described membrane lipid, most likely enabling the higher stability of the membrane (50). Although the general pattern described previously for other strains was also found in our samples, the influence of a changing nutrient supply on the modulation of the membrane lipid composition has been investigated in only a few studies so far (31). In the current study, temperatures and changing nutrient supplies were examined concurrently. One significant finding is that *M. villosus* revealed a higher total lipid production rate than *M. marburgensis* and *M. okinawensis* under standard growth conditions (127.7 ± 18.5 nmol g^−1^ h^−1^ [*M. villosus*] compared to 27.4 ± 7.0 nmol g^−1^ h^−1^ [*M. okinawensis*] and 17.7 ± 1.2 nmol g^−1^ h^−1^ [*M. marburgensis*]) (Fig. 2). It thus appears that lipidome compositions are less dependent on taxonomy than on environmental conditions.

Among the three strains, *M. okinawensis* revealed the strongest changes in the relative lipid production rates and compositions of lipids at varying incubation temperatures (Fig. 5). Especially, the relative proportions of archaeol versus macrocyclic archaeol were highly dependent on environmental conditions. According to experiments with vesicles and tubules made from synthetic archaeon-type phosphatidylcholines (which resemble archaeol and macrocyclic archaeol), macrocyclization leads to decreased water permeability and increased stability of the vesicles (50). It was suggested that the C-C bond at the methyl ends of the phytanyl chains prevents independent motion and ultimately leads to a more closely packed assembly in the liquid-crystalline state (50). In combination with the results of other lipid culture experiments (28), the assumption was put forward that the incorporation of macrocyclic archaeol could increase overall membrane stability and rigidity and reduce membrane fluidity. Consequently, higher proportions of archaeol than of macrocyclic archaeol would be expected at the lowest temperature, similar to an overall increase in diether lipids over tetraether lipids. However, this does not explain the observation that the rates of production of macrocyclic archaeol are highest at 60°C, the lower boundary of the temperature optimum of *M. okinawensis*.

In *M. villosus*, having its temperature optimum at 80°C, macrocyclic archaeol is produced at a higher rate than archaeol under almost all tested conditions (Fig. 5). Kaneshiro and Clark made a similar observation for Methanocaldococcus jannaschii, a close relative of *M. villosus*, where at higher pressures, more macrocyclic archaeol than archaeol was synthesized (29). So, even though we found macrocyclic archaeol in two strains, there is no general pattern when macrocyclic archaeol is predominating, so we cannot confirm that the macrocyclic archaeol production rate is highest at low temperatures and high pressure.

*M. marburgensis* produced GDGTs and GMGTs, with additional methylations in the alkyl chains, but no macrocyclic archaeol. GDGT and GMGT proportions varied with changing temperatures. The highest degree of methylation was found at 60°C for both GDGTs and GMGTs (see reference 37). This result is in contrast to those of another study (51), where a higher percentage of methylated lipids at 45°C than at 60°C and 70°C for GDGTs and GMGTs was reported for M. thermautotrophicus, a close relative of *M. marburgensis*. The authors of that study further observed distinctly higher contents of nonmethylated GDGT-0a and GMGT-0a than those with one (GDGT-0b and GMGT-0b) and two (GDGT-0c and GMGT-0c) extra methylations, whereas in the present study, almost equal amounts of GDGT-0a and GDGT-0b and a predominance of GMGT-0b′ over GMGT-0a′ and GMGT-0c′ at all temperatures were observed.

In contrast, our results confirm the findings of the above-mentioned study (51), where a higher proportion of methylated homologs prevailed among the GMGTs than among the GDGTs at all three chosen temperatures. Another study showed a predominance of GDGT-0b, -c, and -d compared to the nonmethylated GDGT-0a in *M. thermautotrophicus* grown at 65°C (31). When comparing the results of these three studies, it has to be considered that in all experiments different growth temperatures were chosen. It is thus still an open question what triggers the expression of varying lipid patterns at different temperatures in *M. marburgensis*.

### Methanogens produce lipids and amino acids of biotechnological interest.

As the world strives for a cleaner and more sustainable future, biotechnology- and microbe-based processes will become increasingly important. Today, microbes are already a fundamental component of the industry, where they are used to produce a wide range of high-value compounds such as pharmaceuticals, alcohols, fuels, and vital precursors for industrial processes (13). Although most established production processes for amino acids are based on bacteria or yeasts, archaea are of great interest for such applications due to their unique physiologies (3, 9) and enzymes (52). Here, we demonstrate that the biotechnological potential of *M. marburgensis*, *M. villosus*, and *M. okinawensis* goes beyond CH_4_ production. By controlling the environmental conditions, archaeal production of high-value compounds such as isoprenoid lipids and proteinogenic amino acids becomes feasible, which would allow the implementation of these revenue streams in addition to biomethanation. Moreover, the shift in the CH_4_/H_2_O product ratio might also be of biotechnological relevance as it would allow the detection of temperature deviations, and substrate limitations could then be adjusted to decrease H_2_O production. As shown for *M. marburgensis* and *M. okinawensis*, the average WER/MER ratio is approximately 5% lower at 60°C than at 65°C, but the growth characteristics are almost identical. For *M. okinawensis*, 60°C and 65°C are the lower and upper boundaries of its optimal growth temperature range (34). This finding has implications for both pure-culture and industrial-scale H_2_/CO_2_ biomethanation (9, 11). Therefore, lowering the growth temperature, which also lowers the required (thermal) energy and, consequently, the costs, would have the potential to decrease H_2_O production rates during pure-culture biomethanation. Even if such a small temperature difference does not have an impact on growth, it clearly affects the AAER (Fig. 4) and the lipidome (Fig. 5).

To summarize, this study demonstrates that the levels of excreted amino acids vary between different strains, as the amino acid excretion rate was more than a magnitude lower in *M. marburgensis* than in *M. villosus* and *M. okinawensis*. Furthermore, each species excreted a specific pattern of amino acids. Regarding the lipidome, *M. villosus* showed a higher specific lipid production rate than *M. marburgensis* and *M. okinawensis*, with a particularly strong influence of the incubation temperature. Therefore, bioprocesses using one of the tested strains can now be optimized with regard to CH_4_, amino acid, and lipid production. By varying the temperature, the increased excretion and production of specific amino acids or lipids can be achieved. Furthermore, our study shows that a high gas-to-liquid-volume ratio favors the AAER and the lipid production rate.

Further studies are required in which changing substrate availabilities combined with different temperatures, pH, and pressures will yield more detailed insights into the physiological and biotechnological potentials of methanogens. Studies where the AAER and the lipid production rate are continuously monitored combined with studies on the metabolic pathways will help us to understand the causes of the observed variations and, furthermore, to implement these findings in biotechnological applications. Eventually, the ability to scale up these processes utilizing a low-cost feed (H_2_/CO_2_) as the substrate will bring the use of methanogens closer to industrial commercialization.

## MATERIALS AND METHODS

### Experimental setup.

Methanothermobacter marburgensis DSM 2133, Methanothermococcus okinawensis DSM 14208, and Methanocaldococcus villosus DSM 22612 were obtained from the Deutsche Sammlung von Mikroorganismen und Zellkulturen GmbH (DSMZ) (Braunschweig, Germany). Cultivation was conducted in 120-mL serum bottles (La-Pha-Pack, Langerwehe, Germany) in chemically defined medium (6, 10). The cultivation medium for *M. marburgensis* contained 2.1 g NH_4_Cl, 6.8 g KH_2_PO_4_, 3.6 g Na_2_CO_3_, and 5 mL of a 200× trace element solution and was filled up to 1 L with double-distilled water (ddH_2_O). For *M. villosus* and *M. okinawensis*, DSMZ medium 282 (year 2014) was used, with the following modifications: 0.14 g K_2_HPO_4_ was replaced with 0.183 g K_2_HPO_4_·3H_2_O, and 0.01 g Fe(NH_4_)_2_(SO_4_)_2_·6H_2_O was replaced with 7 mg FeSO_4_·7H_2_O, without the usage of resazurin. The exact procedures for medium preparation and inoculation and the method used were described previously (10). The injected inoculum was taken from the respective precultures in exponential phase and made up 2% of the respective total liquid volumes. The cultures were flushed for approximately 3 s and then gassed once or twice per day with an H_2_/CO_2_ test gas mixture (20 vol% CO_2_ in H_2_) at a relative pressure of approximately 2 × 10^5^ Pa (10). Headspace pressure measurements of the serum bottles were performed using a digital manometer (LEO1-Ei, −1…3 bar; Keller, Germany). For each experimental setup, two independent experiments were performed: one set of quadruplicates without (marked with “I”) and one with (“II”) sampling for OD measurements before each gassing event (according to the method used [10], each time with ~0.7 mL of the sample for OD measurements). Growth was recorded via the OD (wavelength [λ] of 578 nm, blanked with MilliQ water, with a DU800 spectrometer [Beckman Coulter, USA]). No obvious variations in the H_2_O evolution rate (WER) (in millimoles per liter per hour) and the mean CH_4_ evolution rate (MER) (in millimoles per liter per hour) were observed in the two different experimental setups. Also, no general variations in the amino acid excretion pattern and lipidome related to the slowly increasing volume of the gaseous substrates caused by the OD sampling were detected. As we have not observed a large difference in the experiments performed with and those without OD measurements, the data presented are pooled data from the two data sets. However, for completeness, the figures in the supplemental material show the two settings separately.

A negative control, i.e., a bottle that included only medium but was not inoculated, was incubated together with the other bottles as the background reference for the OD and amino acid measurements. Definition of a background reference for the lipid measurements was not feasible due to the small amount of material available after harvesting the zero controls. No eukaryotic or bacterial lipids were detected.

After the completion of each experiment, the biomass and supernatant were harvested by the centrifugation of each culture for 20 min at 4,500 rpm (3,328 relative centrifugal force [rcf]) at 4°C in 50-mL Greiner tubes (Universal 320R; Hettich). Subsequently, cell pellets and three 1-mL samples from the supernatant in each bottle were separately stored in sterile Eppendorf tubes at −20°C until further analysis.

A major part of the experiments was performed at the respective optimal growth temperatures (65°C for *M. marburgensis* and *M. okinawensis* and 80°C for *M. villosus*) and using three liquid volumes, 25, 50, and 75 mL. Furthermore, we performed several complementing experiments depending on the physiology pertaining to these organisms. To examine the influence of the incubation temperature on the lipidome and the amino acid excretion pattern, *M. marburgensis* and *M. okinawensis* were additionally grown at 50°C, 60°C, and 70°C in 50 mL. No growth of *M. marburgensis* was observed at 70°C. For comparison, the hyperthermophilic archaeon *M. villosus* was additionally grown at 65°C, and the volume of the liquid medium (25, 50, and 75 mL) varied. In addition, *M. marburgensis* was grown with a carbonate-free medium, as it was of physiological and biotechnological interest to examine the changes in the lipidome and metabolite patterns in these media. *M. okinawensis* and *M. villosus* are fast-growing organisms (16), which resulted in the gas-limited growth of these strains in our closed batch experimental setting. Therefore, we examined the physiological changes of these organisms under two different gassing regimes: gassing once per day (i.e., approximately every 18 to 25 h) versus twice per day (i.e., every 5 to 15 h) with H_2_/CO_2_ (carbon dioxide) (4:1, vol/vol). An overview of the experimental setup is given in Fig. 1.

### Amino acid and lipid analyses.

Detailed descriptions of the methods and instruments were reported previously (33, 37). All NH_4_^+^ uptake, lipid, and amino acid excretion rates were calculated via endpoint measurements (after harvesting). The only exceptions are the amino acid values from the lysis experiment.

Prior to analysis by gas chromatography (GC)-mass spectrometry (MS) and high-performance liquid chromatography (HPLC)-MS, internal preparation standards were added to 0.1 to 15 mg of freeze-dried biomass. The internal standards used for lipid extraction and analysis were 5-α-cholestane (CAS no. 566 481-21-0) and 1,2-Di-*O*-octadecyl-rac-glycerol (DAGE) C_18:18_ (CAS no. 6076-38-6), both at concentrations of 100 mg/L. The concentration of the internal standard added was adjusted depending on the amount of biomass used for extraction. After the addition of the internal standards, the aliquot was subjected to acid hydrolysis with HCl (10%) at 110°C for 2 h. Subsequently, lipids were extracted four times with *n*-hexane–Dichloromethane (DCM) (4:1, vol/vol) in an ultrasonic bath to obtain the total lipid extract (TLE). An aliquot of the TLE was derivatized with acetic anhydride and pyridine (1:1, vol/vol) at 60°C for 1 h, after which the TLE was stored overnight at room temperature (ca. 20°C) before GC-MS and GC-flame ionization detection (FID) analyses, to derivatize the compounds completely.

A Thermo Scientific Trace Ultra gas chromatograph coupled to a Thermo Scientific DSQ II mass spectrometer was used for compound identification. The GC-FID instruments used were Fisons Instruments GC 8000 series and Fisons Instruments HRGC Mega 2 series instruments. Both instruments were used for the quantification of diether lipids as a control for the HPLC-atmospheric pressure chemical ionization (APCI)-MS measurements (compare the data in references 33 and 37). A detailed description of the GC methods was reported previously (37). In brief, the measurements on the GC-MS instrument were done on a Thermo Scientific Trace GC Ultra gas chromatograph coupled to a Thermo Scientific DSQ II mass spectrometer for identification, whereas the contents were measured with a Fisons Instruments GC 8000 series instrument equipped with a flame ionization detector. The column used for both GC systems was an Agilent HP-5 MS UI fused silica column (length, 30 m; inner diameter [ID], 0.25 mm; film thickness, 0.25 μm). The GC temperature program for both GC-MS and GC-FID was as follows: 50°C for 3 min, 25°C min^−1^ to 230°C with a hold for 2 min, and 6°C for 7 min to 325°C with a final hold for 25 min at 325°C. The response factor between 5-α-cholestane and DAGE C_18:18_ was 1.6:1 on both GC-FID instruments.

Apart from this, archaeal di- and tetraether lipids were determined using a Varian MS Workstation 6.91 HPLC system coupled to a Varian 1200L triple-quadrupole mass spectrometer with an APCI interface operated in positive-ion mode. Separation was achieved on a Grace Prevail Cyano column (150 mm by 2.1 mm, 3-μm particle size) and a guard column of the same material, both held at a temperature of 30°C. The column was used at 30°C. The gradient program used was a linear change from 97.5% *n*-hexane and 2.5% *n*-hexane–2-propanol (90:10, vol/vol) to 75% *n*-hexane and 25% *n*-hexane–2-propanol (90:10, vol/vol) from 0 to 35 min, then linearly to 100% *n*-hexane–2-propanol (90:10, vol/vol) in 5 min with a hold for 8 min, and finally back to 97.5% *n*-hexane and 2.5% *n*-hexane–2-propanol (90:10, vol/vol) for reequilibration of column for 12 min. The solvent flow rate was constant at 0.3 mL min^−1^. C_46_-GDGT (CAS no. 138456-87-8) was added as an internal standard to unfiltered and underivatized dry aliquots of the TLE prior to injection. For HPLC measurements, between 5 and 20% of the total lipid extracts were used for injections. The C_46_ GDGT standard (CAS no. 138456-87-8) (53) was injected at a concentration of 12 mg L^−1^, which is in the same range as the concentrations of the internal standards in the injection vials and those added prior to hydrolysis (see reference 37 for details). The response factors between archaeal di- and tetraether lipids were constantly monitored using a standard mixture, which was injected after every 4 to 5 samples. The standard mixture was prepared from synthetic archaeol (1,2-di-*O*-phytanyl-*sn*-glycerol) (CAS no. 99341-19-2), DAGE C_18:18_, DAGE C_18:18_-4ene (1,3-dilinoleoyl-rac-glycerol) (CAS no. 15818-46-9), and C_46_ GDGT. The response factor between DAGE C_18:18_ and C_46_ GDGT was usually between 1.5:1 and 2:1. The response factor between synthetic archaeol and C_46_ GDGT was usually around 1.5:1. The response factor between the diester DAGE C_18:18_-4ene and C_46_ GDGT was not used for this study. The response factors between C_46_ GDGT and the glycerol dialkyl diethers GDD-0 and GMD-0 could not be determined since there was no standard available. Due to the similar structures, a response factor similar to the one for the tetraether lipids was assumed. Hence, the concentrations of GDD-0 and GMD-0 may include errors and must be taken with caution.

Structural identification of archaeal di- and tetraether lipids was conducted using HPLC-APCI-tandem MS (MS/MS) (37). Aliquots of the TLEs were dissolved in *n*-hexane–2-propanol (99:1, vol/vol) to a concentration of 1 mg mL^−1^ and filtered through a 0.45-μm polytetrafluoroethylene (PTFE) filter prior to analysis. Normal-phase HPLC was conducted using a Waters Alliance 2690 HPLC system fitted with a Grace Prevail Cyano column (150- by 2.1-mm ID, 3-μm particle size) and a security guard column cartridge of the same material. MS/MS experiments were done using a Micromass Quattro LC triple-quadrupole mass spectrometer equipped with an APCI interface operated in positive-ion mode. Structural identification of archaeal lipids was done by comparison with published mass spectra (51, 54–56). Chromatograms showing the distribution of the archaeal lipids are provided in Fig. S4 in the supplemental material (see also references 37 and 51).

10.1128/msystems.01159-22.4 FIG S4 Representative HPLC-APCI-MS chromatogram of Methanothermobacter marburgensis showing the distribution of GDGTs and GMGTs. Shown are the total ion chromatograms from *m/z* 950 to 1,500 and the indicated mass chromatograms (*m/z* 1,302.3, GDGT-0a; *m/z* 1,316.3, GDGT-0b; *m/z* 1,330.4, GDGT-0c; *m/z* 1,300.3, GMGT-0a [both isomers]; *m/z* 1,314.3, GMGT-0b [both isomers]; *m/z* 1,328.3, GMGT-0c [both isomers]). *M. marburgensis* was grown at 60°C in 50 mL with OD measurements, but the basic lipid pattern was the same under all conditions. Download FIG S4, PDF file, 0.1 MB. Copyright © 2023 Taubner et al. 2023 Taubner et al. https://creativecommons.org/licenses/by/4.0/ This content is distributed under the terms of the Creative Commons Attribution 4.0 International license.

As the incubation time varied from one experiment to the other, the amino acid and lipid concentrations were normalized by dividing by the respective total incubation times. The obtained values are described as AAERs and (specific) lipid production rates, respectively. The values for the amino acids cysteine, selenocysteine, pyrrolysine, and proline were below the detection limit.

For half of the experiments with *M. villosus*, i.e., all with combinations of 65°C with twice-per-day gassing and 80°C with once-per-day gassing, the concentrations were determined using 5-α-cholestane and C_46_ GDGT instead of DAGE C_18:18_ and C_46_ GDGT. Quantification with 5-α-cholestane led to 20%-higher specific lipid production rates than by quantification with DAGE C_18:18_. This was corrected by applying a compensation factor of 0.8.

### Dry weight determination.

All organisms were grown to a minimal optical density at 578 nm (OD_578_) of 0.3 in serum flasks. Ten milliliters of undiluted and diluted (1:2, 1:3, 1:4, 1:5, 1:7, 1:8, and 1:10) samples in quadruplicates (*n* = 4) was pipetted onto a 0.22-μm Durapore membrane filter (Merck Millipore, MA, USA) connected to and vacuumed on a glass filtering apparatus (Duran, Wertheim, Germany). Filters were dried overnight at 75°C and weighed before and after the addition of biomass. The biomass (milligrams) was plotted against the OD_578_ values. The resulting k-value was used as the factor to calculate biomass (in grams per liter) (10). The k-values were 0.36 for *M. marburgensis*, 0.49 for *M. okinawensis*, and 0.31 for *M. villosus*. The k-values in combination with the OD_578_ values were used for the calculation of the viable and unviable biomasses (in grams per liter) in the course of the lysis and fluorescence-activated cell sorting (FACS) experiments (see Fig. S2 in the supplemental material).

### Fluorescence-activated cell sorting measurement.

For cell viability analysis, FACS measurement was applied. Briefly, 20 μL of an actively growing culture was diluted with 400 μL of the respective minimal media, stained with Syto9 and propidium iodide (PI) (Live/Dead BacLight bacterial viability kit; Invitrogen), and analyzed (FACSCanto II; BD) (Syto9, fluorescein isothiocyanate [FITC]; PI, peridinin chlorophyll protein [PerCP]-Cy5-5H). Fluorescence compensation was performed using BD FACSDiva software. For each measurement, 10,000 events were recorded and gated (Fig. S1). Cell counts were performed as previously described (57). Total amino acid contents and dead/live biomass contents of *M. marburgensis*, *M. okinawensis*, and *M. villosus* are given in Fig. S2.

### NH_4_^+^ uptake determination.

NH_4_^+^ uptake determination was performed using a modified procedure described previously (58). The oxidation solution, the color reagent, and the NH_4_Cl stock solution were prepared freshly before measurements. As standards, nine different concentrations ranging from 100 μmol L^−1^ to 1,000 μmol L^−1^ of NH_4_Cl were prepared. Samples were diluted with MilliQ water to a final concentration between the standard ranges. Before measurements, 300 μL of color reagent and 120 μL of oxidation solution were added immediately to the standards and samples, which were then mixed briefly. After 30 min in the dark, standards and samples were measured at 660 nm on a 96-well plate (Microtest plate 96 well, F; Sarstedt AG & Co., Nümbrecht, Germany) with a plate photometer (Sunrise plate reader; Tecan Group AG, Männedorf, Switzerland). The regression curve always had an *R*^2^ value of >0.999. Results are presented in Fig. S2 and S3. Here, the value of the zero control was always subtracted to receive the actual NH_4_^+^ uptake (rate).

### Statistical analysis.

Outliers in the data set were identified and excluded when the respective values showed a deviation of more than 100% relative to the mean value of the other replicates from the same experimental set. When two (or more) values showed this characteristic, none were excluded. Furthermore, for some single amino acids or lipids, a value was not determinable. Some of the single values were negative. Negative values indicate that the concentration measured in the negative control was higher than that in the respective replicate (bottle). These negative values were neglected when calculating the total number. The total values presented in the figures were calculated as the mean values for the total amino acids or lipids from every single replicate (bottle) of the respective experiment.

### Data availability.

The comprehensive collection of the raw data has been deposited in the Phaidra online repository (https://doi.org/10.25365/phaidra.380) (59).
