# Supplementary figures and images for: Lipidomics and Comparative Metabolite Excretion Analysis of Methanogenic Archaea Reveal Organism-Specific Adaptations to Varying Temperatures and Substrate Concentrations
Source: mSystems. 2023 Mar 7;8(2):e01159-22. doi: 10.1128/msystems.01159-22 (PMC10134847; doi:10.1128/msystems.01159-22)

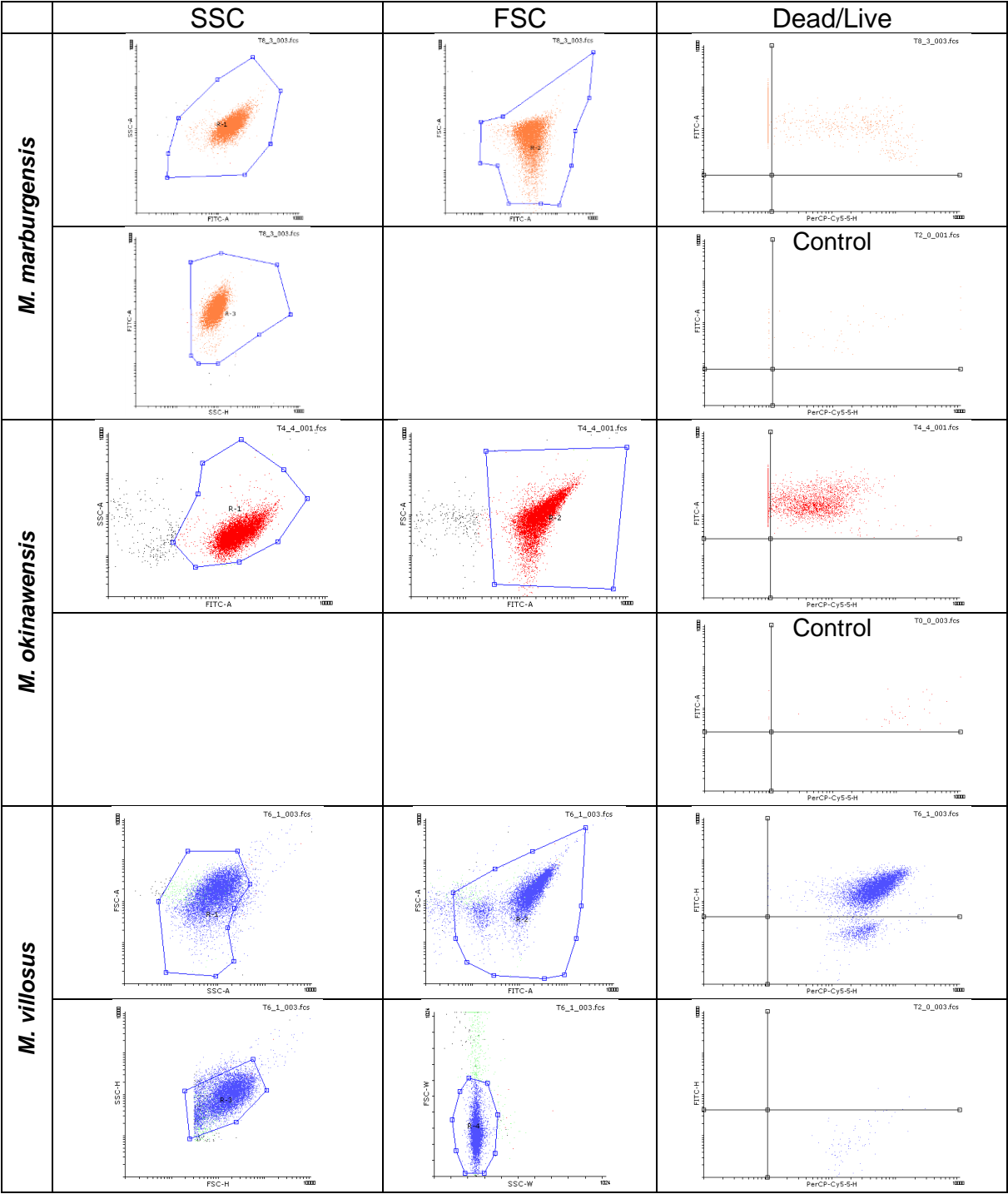

Figure S1, page 1/1

Supplement: FIG S1 [file msystems.01159-22-s0001.pdf]

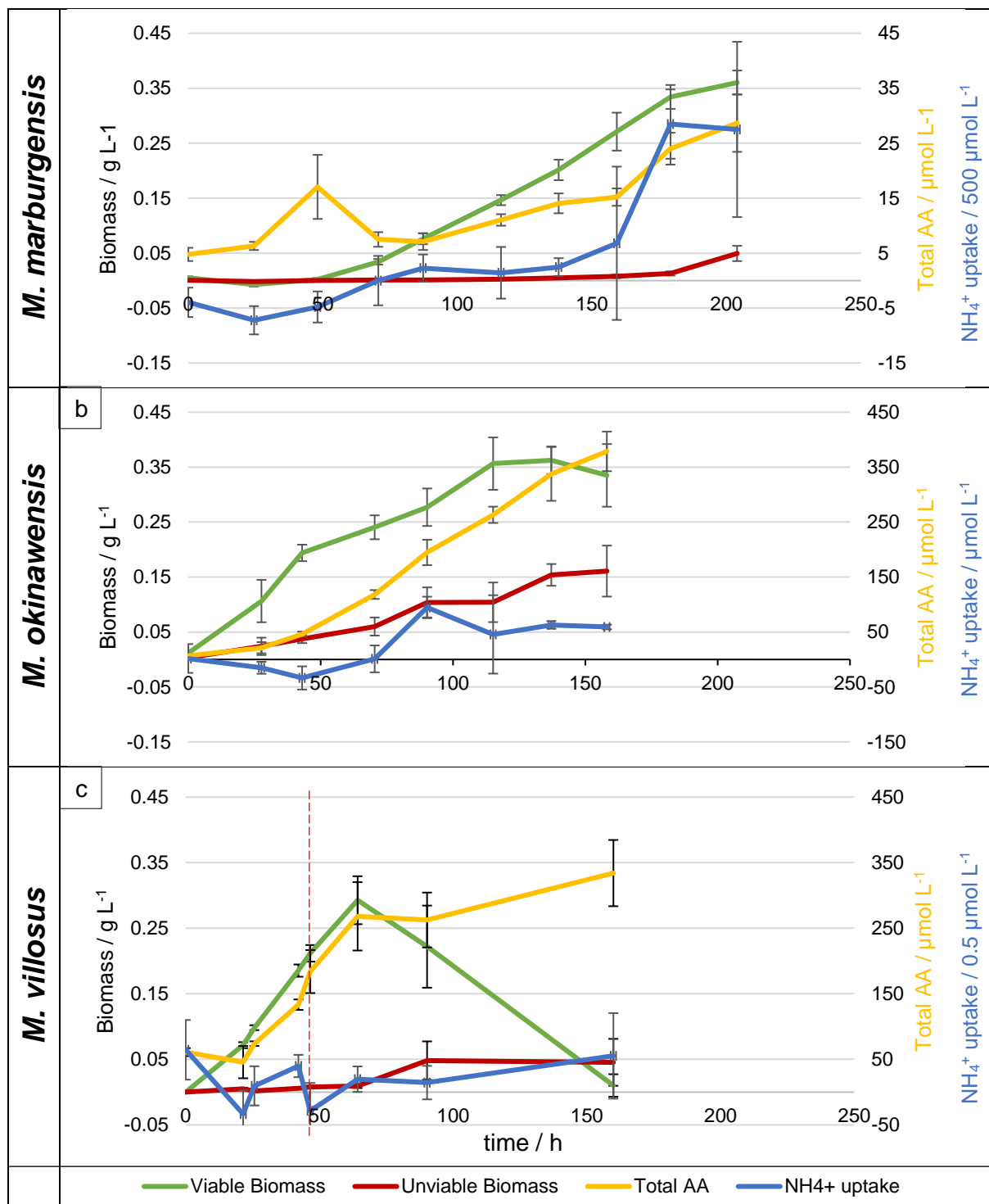

Figure S2, page 1/1

Supplement: FIG S2 [file msystems.01159-22-s0002.pdf]

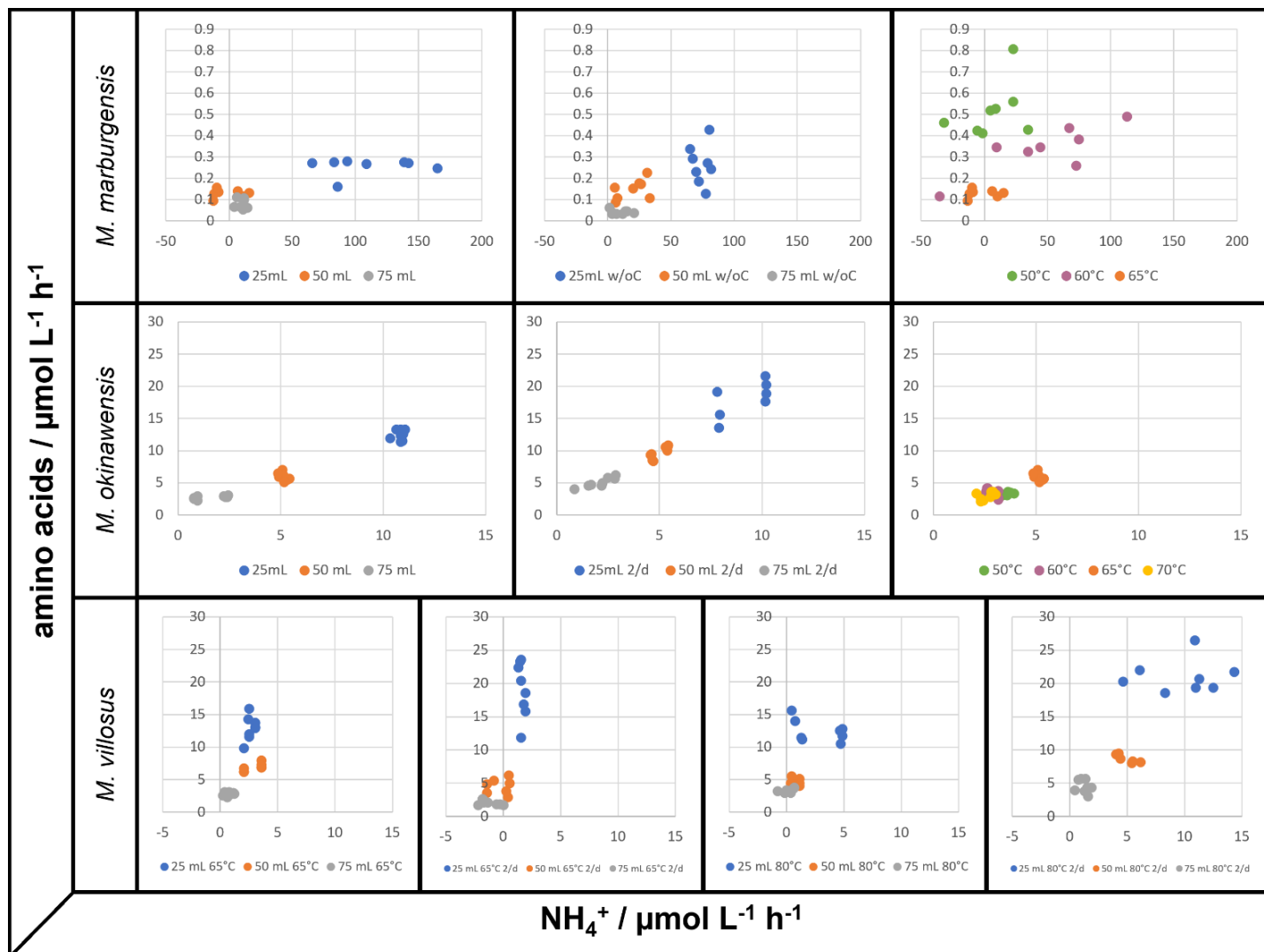

Figure S3, page 1/1

Supplement: FIG S3 [file msystems.01159-22-s0003.pdf]

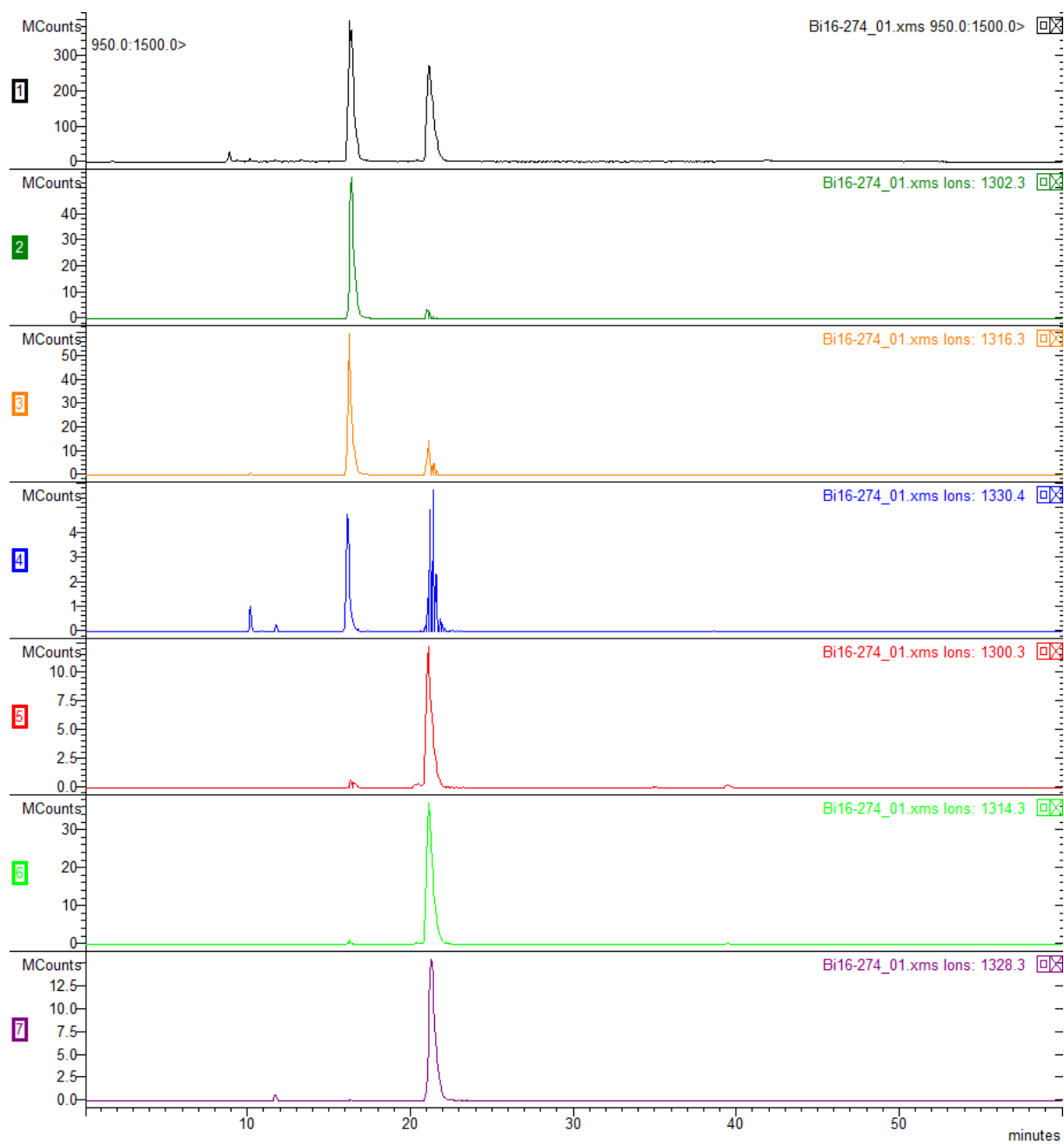

Figure S4, page 1/1

Supplement: FIG S4 [file msystems.01159-22-s0004.pdf]
